# Sex-specific differences in the mechanisms for enhanced thromboxane A_2_-mediated vasoconstriction in adult offspring exposed to prenatal hypoxia

**DOI:** 10.1186/s13293-024-00627-x

**Published:** 2024-06-19

**Authors:** Murilo E. Graton, Floor Spaans, Rose He, Paulami Chatterjee, Raven Kirschenman, Anita Quon, Tom J. Phillips, C. Patrick Case, Sandra T. Davidge

**Affiliations:** 1 Department of Obstetrics and Gynecology, University of Alberta, Edmonton, AB T6G 2R3 Canada; 2 Women and Children’s Health Research Institute, University of Alberta, Edmonton, AB T6G 2R3 Canada; 3 Department of Physiology, University of Alberta, Edmonton, AB T6G 2R3 Canada; 4 UK Dementia Research Institute, Cardiff University, Cardiff, W1T 7NF UK; 5 Musculoskeletal Research Unit, University of Bristol, Bristol, BS8 1QU UK

**Keywords:** Pregnancy complications, Prenatal hypoxia, Developmental origins of health and disease, Placental treatment, Coronary arteries, Mesenteric arteries, Thromboxane A_2_, Nitric oxide, nMitoQ, Sex differences

## Abstract

**Background:**

Prenatal hypoxia, a common pregnancy complication, leads to impaired cardiovascular outcomes in the adult offspring. It results in impaired vasodilation in coronary and mesenteric arteries of the adult offspring, due to reduced nitric oxide (NO). Thromboxane A_2_ (TxA_2_) is a potent vasoconstrictor increased in cardiovascular diseases, but its role in the impact of prenatal hypoxia is unknown. To prevent the risk of cardiovascular disease by prenatal hypoxia, we have tested a maternal treatment using a nanoparticle-encapsulated mitochondrial antioxidant (nMitoQ). We hypothesized that prenatal hypoxia enhances vascular TxA_2_ responses in the adult offspring, due to decreased NO modulation, and that this might be prevented by maternal nMitoQ treatment.

**Methods:**

Pregnant Sprague–Dawley rats received a single intravenous injection (100 µL) of vehicle (saline) or nMitoQ (125 µmol/L) on gestational day (GD)15 and were exposed to normoxia (21% O_2_) or hypoxia (11% O_2_) from GD15 to GD21 (term = 22 days). Coronary and mesenteric arteries were isolated from the 4-month-old female and male offspring, and vasoconstriction responses to U46619 (TxA_2_ analog) were evaluated using wire myography. In mesenteric arteries, L-NAME (pan-NO synthase (NOS) inhibitor) was used to assess NO modulation. Mesenteric artery endothelial (e)NOS, and TxA_2_ receptor expression, superoxide, and 3-nitrotyrosine levels were assessed by immunofluorescence.

**Results:**

Prenatal hypoxia resulted in increased U46619 responsiveness in coronary and mesenteric arteries of the female offspring, and to a lesser extent in the male offspring, which was prevented by nMitoQ. In females, there was a reduced impact of L-NAME in mesenteric arteries of the prenatal hypoxia saline-treated females, and reduced 3-nitrotyrosine levels. In males, L-NAME increased U46619 responses in mesenteric artery to a similar extent, but TxA_2_ receptor expression was increased by prenatal hypoxia. There were no changes in eNOS or superoxide levels.

**Conclusions:**

Prenatal hypoxia increased TxA_2_ vasoconstrictor capacity in the adult offspring in a sex-specific manner, via reduced NO modulation in females and increased TP expression in males. Maternal placental antioxidant treatment prevented the impact of prenatal hypoxia. These findings increase our understanding of how complicated pregnancies can lead to a sex difference in the programming of cardiovascular disease in the adult offspring.

**Graphical Abstract:**

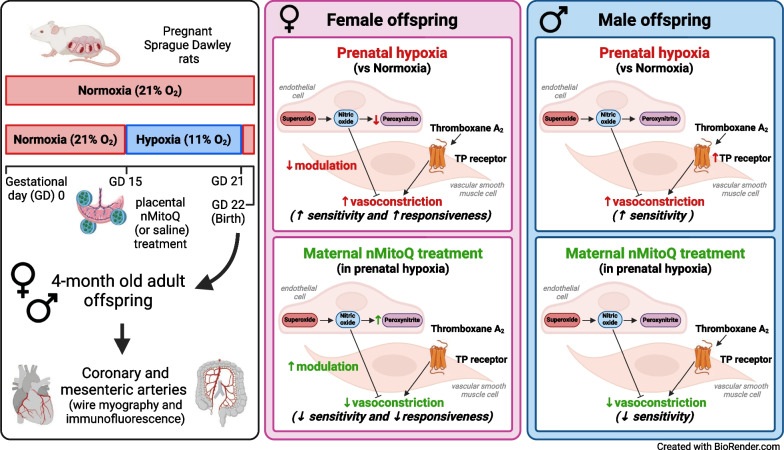

## Highlights

- Prenatal hypoxia increases thromboxane-mediated vasoconstrictor responses in both coronary and mesenteric arteries from the adult female and male offspring. This was more pronounced in females, and was prevented by maternal placental antioxidant treatment in pregnancy.
- The mechanisms for the increased thromboxane-mediated vasoconstrictor responses by prenatal hypoxia exposure were sex-specific, where females had reduced nitric oxide modulation of vasoconstriction, while males had increased thromboxane receptor expression.
- There are sex-specific differences on how prenatal hypoxia impairs vascular function in the adult offspring. Placenta-targeted therapies in pregnancy should be considered to improve cardiovascular outcomes in the offspring later life but should take the sex of the fetus into consideration.

## Plain language summary

Prenatal hypoxia, when the fetus does not receive enough oxygen, is a common problem during pregnancy that impacts the developing fetus. It is associated with an increased risk of cardiovascular disease in the offspring in adulthood. While the mechanisms are not fully understood, the blood vessel function in the offspring may be impacted by prenatal hypoxia. We hypothesize that prenatal hypoxia increases the constriction of the blood vessels in the offspring. The placenta, an essential organ for fetal development, supplies oxygen and nutrients to the fetus. In prenatal hypoxia pregnancies, the placenta does not work properly. We have been studying a placental treatment (called nMitoQ) to improve placenta function and thereby the blood vessel function of the offspring. We used a rat model of prenatal hypoxia, where pregnant rats (dams) were placed in a low oxygen environment (hypoxia) during the last trimester of pregnancy. Control rats were kept in normal oxygen conditions. The dams were treated with nMitoQ, or with saline (control). Next, we studied the blood vessels of the offspring in adulthood. We found that prenatal hypoxia increases the constriction of the blood vessels, which was prevented by treating the dams with nMitoQ. Interestingly, this impact was more severe in females compared to males, and the mechanisms were different between the sexes. This study helps in the understanding of how complicated pregnancies can impair cardiovascular health in the offspring, and in a potential development of targeted and sex-specific therapies for those offspring at high risk for future cardiovascular disease.

## Background

Cardiovascular diseases (CVDs) are the leading cause of mortality worldwide [1]. The knowledge and the detection of risk factors preceding CVDs is essential to identify susceptible individuals and to apply intervention strategies to address these risk factors [2]. Classical risk factors for CVDs include hypertension, diabetes, obesity, dyslipidemia, smoking, and sedentary behaviour [3]. However, there is now a growing body of evidence that complications during pregnancy can contribute to the development of CVDs in the offspring [4], a growing field of study termed the developmental origins of health and disease [reviewed in [5–11]]. Prenatal hypoxia is one of the most common consequences of many pregnancy complications (such as preeclampsia) [12]. Prenatal hypoxia may be the consequence of functional and morphologic changes in the placenta [13, 14] and has been shown to impair fetal development [15]. Consequently, exposure to prenatal hypoxia in utero is a significant risk factor for the development of metabolic and cardiovascular disease in the offspring in adult life [16]. However, the mechanisms are not fully understood.

We have previously demonstrated that prenatal hypoxia impairs vascular function in the offspring. For example, prenatal hypoxia impaired vasodilation responses in coronary arteries [17] and mesenteric arteries [18] of the adult male and female offspring. In addition, vasoconstrictor responses to big-endothelin 1 were augmented in mesenteric arteries from aged male, but not female, prenatal hypoxia offspring [19]. This appeared to be (partly) due to a reduction in the vasodilator molecule nitric oxide (NO), an important vasodilation molecule that is produced by NO synthase (NOS) and induces vasodilation and can modulate smooth muscle constriction [20]. The endothelium plays an important role as key regulator of vascular homeostasis by the production of several vasoactive relaxing (such as NO) and constricting molecules [21, 22]. However, in conditions of oxidative stress, NO bioavailability may be reduced, as NO can be scavenged by reactive oxygen species (ROS) such as superoxide, to form peroxynitrite [23]. This can result in altered NO modulation of vascular responses as NO is a major regulator of the vascular tone and vascular homeostasis [24].

Thromboxane A_2_ (TxA_2_) is an eicosanoid mediator produced in prostaglandin H synthase (PGHS) pathway using arachidonic acid as substrate [25], and a potent vasoconstrictor due to the activation of thromboxane prostanoid (TP) receptors in smooth muscle cells [26]. Increased formation of TxA_2_ has been implicated in the pathogenesis of various cardiovascular diseases [25]. Indeed, in male adult offspring, exposure to prenatal hypoxia was reported to increase the sensitivity to TxA_2_ in mesenteric arteries, however, female offspring were not assessed [27]. In addition, we recently showed that the impaired vasodilation responses in coronary arteries of prenatal hypoxia male and female offspring appeared to be PGHS-mediated [17]. As PGHS is the enzyme upstream from TxA_2_ production, this could suggest there may be changes in the vascular eicosanoid/TxA_2_ signaling pathway in mesenteric and coronary arteries of prenatal hypoxia offspring. However, the impact of prenatal hypoxia on TxA_2_ responsiveness in the coronary arteries of the adult offspring, and mesenteric arteries of the female adult offspring, remains to be investigated. Moreover, it is not known if the reduced NO bioavailability could also lead to a lower NO modulation of vasoconstriction.

Placental insufficiency, likely due to oxidative stress by the overproduction of reactive oxygen species (ROS) by the mitochondria [28], can adversely affect the development and function of the placenta leading to fetal hypoxia and adverse health outcomes for both mother and fetus [29]. Because of this detrimental role of oxidative stress in the placenta, there has been a surge of interest in the development of placenta-targeted therapies to improve fetal and offspring outcomes in complicated pregnancies [30]. Therefore, our lab has been assessing the effects of a maternal treatment with a mitochondrial antioxidant, MitoQ, encapsulated into nanoparticles (nMitoQ) [31–35]. Due to their zeta potential (− 20 mV) and diameter (180 nm) the nanoparticles accumulate in the placenta [32, 36]. We previously reported that nMitoQ did not to cross the placenta and did not have clear adverse effects on the fetus [36, 37]. We also showed that maternal nMitoQ treatment in prenatal hypoxia offspring reduced male and female placental superoxide levels [32] and oxidative stress, and improved placental mitochondrial function [33]. Moreover, maternal nMitoQ treatment in prenatal hypoxia pregnancies improved cardiac tolerance to ischemia/reperfusion (I/R) insult in both male and female adult offspring [34], prevented cardiac diastolic dysfunction in females [31], and increased cardiac mitochondrial function in the female adult offspring [35]. Lastly, we also showed that maternal nMitoQ treatment improved endothelium-dependent vasorelaxation in mesenteric arteries from both male and female aged offspring [31]. However, if maternal nMitoQ treatment can prevent the impact of prenatal hypoxia on TxA_2_ responsiveness in coronary and mesenteric arteries from the adult offspring is not known. In the current study, we hypothesized that the exposure to prenatal hypoxia increases vasoconstriction to TxA_2_ in coronary and mesenteric arteries of the male and female adult offspring, and that this is prevented by maternal nMitoQ treatment in pregnancy.

## Methods

### Animal model of prenatal hypoxia

Female and male (for breeding) Sprague Dawley rats (12 weeks old) were purchased from Charles River Laboratories (Kingston, NY, USA), and were housed in a controlled environment with 14:10 h light–dark cycles and room temperature of 22 ± 1 °C. Rats were acclimatized for at least one week and had ad libitum access to a standard chow diet and water. Female rats were mated overnight with a male, and the presence of sperm in a vaginal smear was considered gestational day (GD) 0 (term pregnancy = GD22). Dams were single housed during pregnancy. On GD15, pregnant dams received a single tail vein injection of either saline (100 μL) or nMitoQ (100 μL of a 125 μmol/L solution). Since unloaded nanoparticles were previously shown to be inert, physiological saline solution was used as a control [36]. The nMitoQ solution was prepared as described previously by Phillips et al*.* [36], and the experimental design is based on previous studies from our laboratory [31, 34, 37]. From GD15–21, rats were exposed to hypoxia (p-Hypoxia, 11% O_2_) or were kept under normoxic conditions (Normoxia, 21% O_2_) (n = 7–14 dams per group). On GD21, dams were removed from the hypoxic chamber and allowed to give birth in standard housing and oxygen conditions. To standardize postnatal conditions, where possible, litter size was decreased at birth to 8 pups/litter (4 males and 4 females). On postnatal day 21, the offspring were weaned, sex-matched, and double housed at standard housing conditions until 4 months of age.

At 4 months of age, the male and female adult offspring were anesthetized with isoflurane (4% in 100% O_2_). The heart and the mesenteric arcade were immediately excised and placed in ice-cold HEPES-buffered physiological saline solution (PSS; in mmol/L: 10 HEPES, 5.5 glucose, 1.56 CaCl_2_, 4.7 KCl, 142 NaCl, 1.17 MgSO_4_, 1.18 KH_2_PO_4_, and pH 7.4). Left anterior descending coronary arteries (cardiac conduit artery responsible for ~ 50% of the total cardiac blood supply; ~ 150–250 µm) and second order mesenteric arteries (systemic resistance arteries that regulate blood pressure; ~ 150–250 µm) were isolated and cleaned from surrounding fat tissues for assessment of ex vivo vascular function using wire myography. Segments of the mesenteric arteries were snap-frozen in optimal cutting temperature compound (Tissue-Tek®, Sakura Finetek, Torrance, USA), cut into 9 μm-thick cryosections, thaw-mounted on super-frost slides, and stored at − 80 °C for immunofluorescence assays. As the left anterior descending coronary arteries are very short, all tissues were used for myography and no segments were frozen for molecular analysis.

### Assessment of vascular function by wire myography

Isolated coronary and mesenteric arteries were cut into 2 mm segments and mounted on a wire myograph system (620 M DMT, Copenhagen, Denmark) using 40 μm tungsten wires, and data were recorded using the LabChart software (version 8.1.13; AD Instruments; Colorado Springs, USA). According to previously established protocols for rat coronary arteries [17] and rat mesenteric arteries [38], vessels were normalized to an optimal resting tension through incremental increases of their diameter to 100 mmHg in transmural pressure. After a 20-min equilibration period in PSS at 37 °C, endothelium and vascular smooth muscle functionality was tested with two doses of phenylephrine (PE; 10 µmol/L) and one dose of methylcholine (MCh; 3 µmol/L). Vessels were then washed and allowed to rest for 30 min. Next, cumulative concentration response curves (CCRCs) to 9,11-dideoxy-9αm11α-methanoepoxy prostaglandin F_2α_ (U46619), a TxA_2_ analog [39], were performed (0.1 nmol/L – 10 µmol/L, added in 2 min intervals, or until plateau). In the mesenteric arteries, to determine the role of NO modulation in the thromboxane-mediated constriction CCRCs to U46619 were performed in the absence (control) or in the presence of the pan-NOS inhibitor Nω-nitro-L-arginine methyl ester (L-NAME, 100 µmol/L, added 30 min prior to the start of the CCRC). Due to tissue limitations of the left anterior descending coronary arteries (one ~ 2 mm segment per animal), we were not able to perform mechanistic studies using inhibitors in the coronary arteries. After the CCRC to U46619, all vessels were allowed to rest for 15 min before high potassium physiological solution (KPSS 123 mmol/L) was added to the baths to assess maximum smooth muscle contractility.

### Mesenteric artery endothelial nitric oxide synthase, 3-nitrotyrosine, and TP receptor expression by immunofluorescence staining

Mesenteric artery cryosections were thawed, fixed in cold acetone (− 20 °C, 10 min), treated with sodium borohydride (1 mg/mL) in phosphate buffered saline (PBS, pH 7.4) for 20 min to quench autofluorescence, and thrice washed in PBS for 10 min. The sections were then incubated with a blocking solution (2% bovine serum albumin (BSA) in PBS, 5% donkey serum, and 0.01% Triton-X) at room temperature for 1.5 h, followed by three washes with PBS. The sections were then incubated in a humid chamber overnight at 4 °C with a rabbit polyclonal anti-NOS3 (N-20) (sc-653, Santa Cruz Biotechnology, Santa Cruz, USA; 1:100 dilution in 2% BSA in PBS) or anti-nitrotyrosine (A21285, Invitrogen, Waltham, USA; 1:100 dilution in 2% BSA in PBS) or anti-thromboxane A_2_ receptor (101882, Cayman Chemical, USA; 1:100 dilution in 2% BSA in PBS). The next day, slides were washed with PBS (3x) and incubated with donkey (for eNOS and TP receptor; Alexa Fluor™ 546, A11040, Invitrogen, USA; 1:250 dilution in 2% BSA in PBS) or goat (for 3-nitrotyrosine; Alexa Fluor™ 546, A11035, Invitrogen, USA; 1:250 dilution in 2% BSA in PBS) anti-rabbit IgG secondary antibody with 1:500 dilution in 2% BSA of Hoechst 33342 (Invitrogen), for 1 h at room temperature in a humid chamber. After the final PBS washes (3x), the slides were then mounted with Vectashield® Antifade Mounting Medium with DAPI (H-1200 solution, Vector Laboratories Inc., Newark, USA), coverslipped and sealed with clear nail polish, stored in the dark and left to dry overnight. Images of the mesenteric arteries were taken the following day using confocal microscopy.

### Levels of vascular reactive oxygen species

ROS levels in mesenteric arteries were assessed using dihydroethidium (DHE) fluorescent staining. In short, mesenteric artery cryosections were first incubated in Hank's Balanced Salt Solution (HBSS) at 37 °C for 10 min followed by an incubation with a dihydroethidium (DHE) fluorescent probe (10057; Biotium, USA; 1:1000 dilution, in 1:500 dilution of Hoechst 33342 in HBSS) was added and incubated at 37 °C for 30 min. Slides were then washed with HBSS (3x), coverslipped, and imaged immediately using confocal microscopy.

### Image analysis

All images (eNOS, ROS, nitrotyrosine, and TP receptor) were captured using a confocal microscope (Zeiss LSM 700, Germany) coupled to the Zen Black image processing software (8.1, Zeiss). Images were analyzed using ImageJ software (ImageJ 2.9.0), as previously described by our group [40, 41]. One section of mesenteric artery per offspring per sex per dam was used for the analysis. The mean fluorescence intensity (MFI, arbitrary units) of each image was then normalized to the average fluorescence of the normoxia saline group, which was taken as the baseline value.

### Statistical analysis

**Fig. 1:**
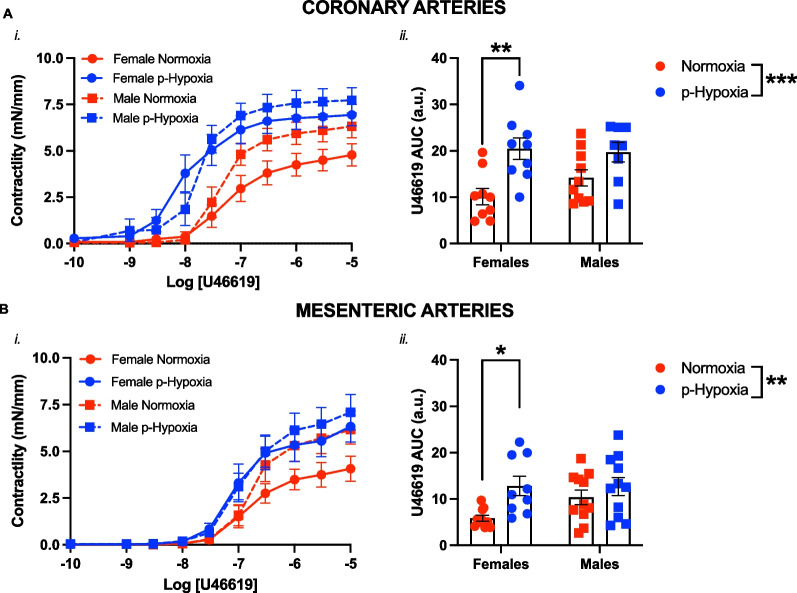
Sex differences in thromboxane A_2_-mediated vasoconstriction in coronary and mesenteric arteries of prenatal hypoxia adult offspring. Cumulative concentration response curves to the thromboxane A_2_ analog U46619 (*i*) in coronary (**A**) and mesenteric arteries (**B**) in female (circles) and male (squares) offspring exposed to normoxia (red) or hypoxia (blue; p-Hypoxia) during pregnancy. Data in (*i*) are summarized as area under the curve (AUC; *ii*). Data are presented as mean ± SEM and were analyzed with two-way ANOVA with Sidak’s multiple comparisons post-hoc test (*p < 0.05; **p < 0.01; ***p < 0.001). N = 7–14 offspring/n = 1 offspring per dam/group

**Fig. 2:**
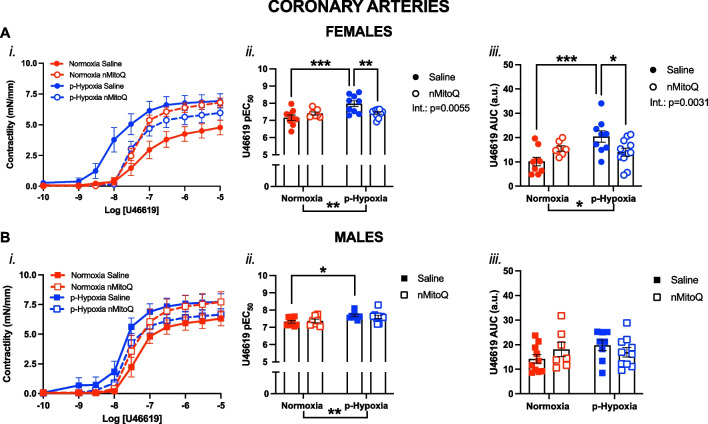
Thromboxane A_2_-mediated vasoconstriction in *coronary arteries* of female and male adult offspring exposed to prenatal hypoxia. Cumulative concentration response curves to the thromboxane A_2_ analog U46619 (*i*) in coronary arteries in female (**A**, circles) and male (**B**, squares) offspring exposed to normoxia (red) or hypoxia (blue; p-Hypoxia) during pregnancy, and after receiving maternal treatment with saline (vehicle, closed circles) or nMitoQ (open circles). Data in (*i*) are summarized as the negative logarithm of the half-maximal effective concentration (pEC_50_; *ii*) and as the area under the curve (AUC; *iii*). Data are presented as mean ± SEM and were analyzed with two-way ANOVA with Sidak’s multiple comparisons post-hoc test (*p < 0.05; **p < 0.01; ***p < 0.001). N = 7–11 offspring/ n = 1 offspring per dam/group. *a.u.* arbitrary units

**Fig. 3:**
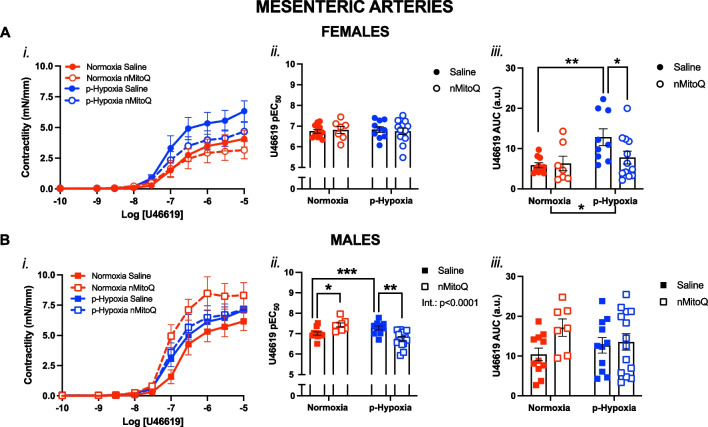
Thromboxane A_2_-mediated vasoconstriction in *mesenteric arteries* of female and male adult offspring exposed to prenatal hypoxia. Cumulative concentration response curves to the thromboxane A_2_ analog U46619 (*i*) in mesenteric arteries in female (**A**, circles) and male (**B**, squares) offspring exposed to normoxia (red) or hypoxia (blue; p-Hypoxia) during pregnancy after receiving maternal treatment with saline (vehicle, closed circles) or nMitoQ (open circles). Data in (*i*) are summarized as the negative logarithm of the half-maximal effective concentration (pEC_50_; *ii*) and as the area under the curve (AUC; *iii*). Data are presented as mean ± SEM and were analyzed with two-way ANOVA with Sidak’s multiple comparisons post-hoc test (*p < 0.05; **p < 0.01; ***p < 0.001). N = 7–14 offspring/ n = 1 offspring per dam/group. *a.u.* arbitrary units

**Fig. 4:**
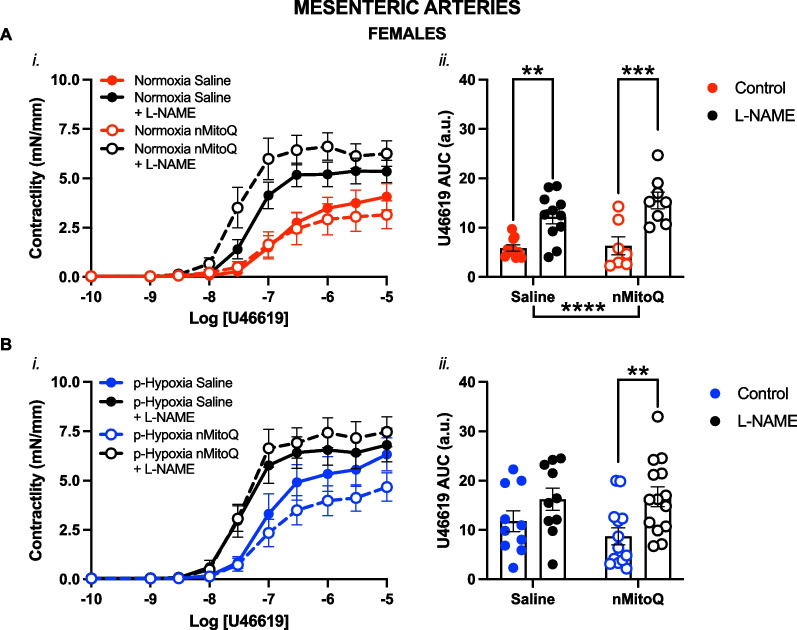
Nitric oxide modulation of thromboxane A_2_-mediated vasoconstriction in mesenteric arteries of prenatal hypoxia female adult offspring. Cumulative concentration response curves to the thromboxane A_2_ analog U46619 (*i*) in the absence or presence of L-NAME (NOS inhibitor, Nω-nitro-L-arginine methyl ester, black symbols) in mesenteric arteries of female offspring exposed to normoxia (red, **A**) or hypoxia (blue, **B**; p-Hypoxia) during pregnancy after receiving maternal treatment with saline (vehicle, closed circles) or nMitoQ (open circles). Data in (*i*) are summarized as area under the curve (AUC; *ii*) and presented as mean ± SEM and were analyzed with two-way ANOVA with Sidak’s multiple comparisons post-hoc test (**p < 0.01, ***p < 0.001, ****p < 0.0001). N = 7–14 offspring/ n = 1 offspring per dam/group. *a.u.* arbitrary units

**Fig. 5:**
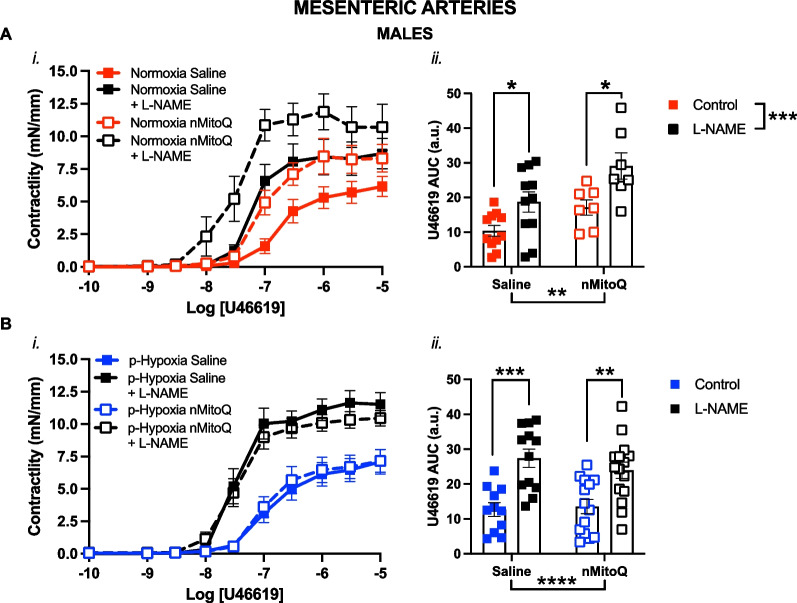
Nitric oxide modulation of thromboxane A_2_-mediated vasoconstriction in mesenteric arteries of prenatal hypoxia male adult offspring. Cumulative concentration response curves to the thromboxane A_2_ analog U46619 (*i*) in the absence or presence of L-NAME (NOS inhibitor, Nω-nitro-L-arginine methyl ester, black symbols) in mesenteric arteries of male offspring exposed to normoxia (red, **A**) or hypoxia (blue, **B**; p-Hypoxia) during pregnancy after receiving maternal treatment with saline (vehicle, closed squares) or nMitoQ (open squares). Data in (*i*) are summarized as area under the curve (AUC; *ii*) and presented as mean ± SEM and were analyzed with two-way ANOVA with Sidak's multiple comparisons post-hoc test (*p < 0.05; **p < 0.01, ***p < 0.001; ****p < 0.0001). N = 7–14 offspring/ n = 1 offspring per dam/group. *a.u.* arbitrary units

**Fig. 6:**
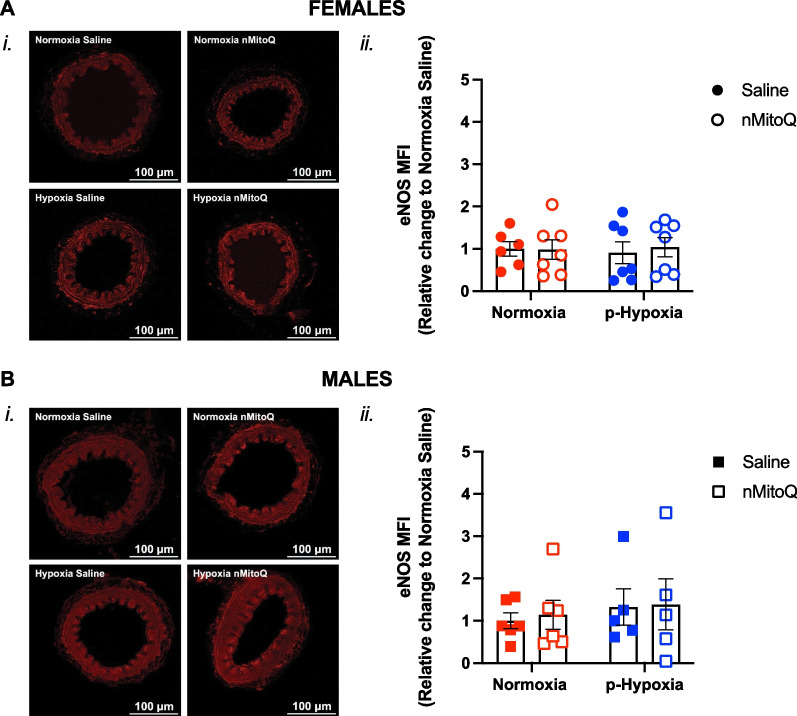
Expression of eNOS in mesenteric arteries of adult offspring exposed to prenatal hypoxia. Representative confocal images (*i*) and quantitative analysis (*ii*) of the mean endothelial nitric oxide synthase (eNOS) expression in mesenteric arteries of female (**A**, circles) and male (**B**, squares) offspring exposed to normoxia (red) or hypoxia (blue; p-Hypoxia) during pregnancy, after receiving maternal treatment with saline (vehicle, closed circles) or nMitoQ (open circles). Data are presented as mean ± SEM and were analyzed with two-way ANOVA with Sidak’s multiple comparisons post-hoc test. N = 5–7 offspring/ n = 1 offspring per dam/group. *a.u.* arbitrary units, *MFI* mean fluorescence intensity

**Fig. 7:**
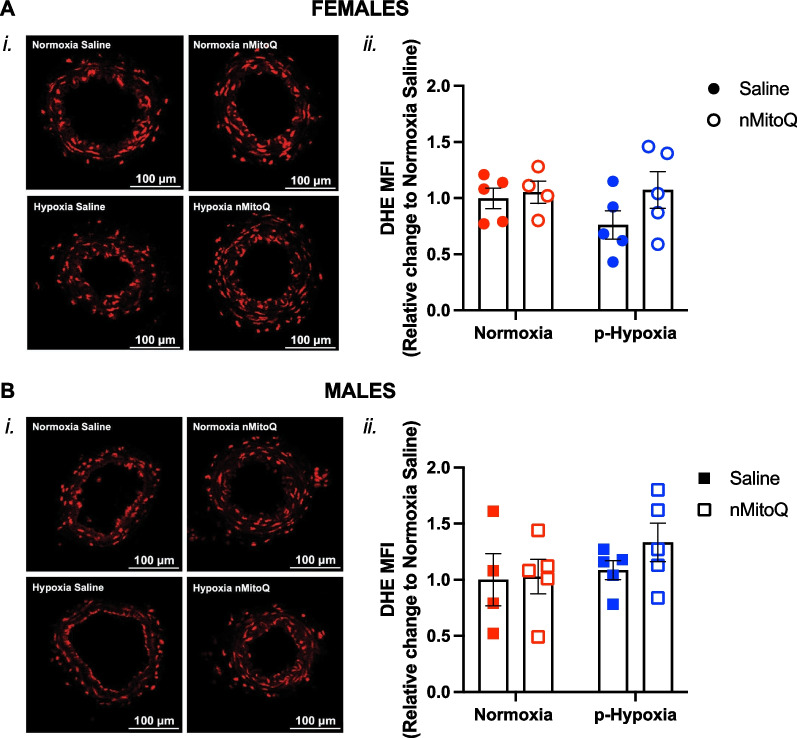
Superoxide detection in mesenteric arteries of adult offspring exposed to prenatal hypoxia. Representative confocal images (*i*) and quantitative analysis (*ii*) of superoxide and other reactive oxygen species, detected by dihydroethidium (DHE) probe, in mesenteric arteries of female (**A**, circles) and male (**B**, females) offspring exposed to normoxia (red) or hypoxia (blue; p-Hypoxia) during pregnancy after receiving maternal treatment with saline (vehicle, closed circles) or nMitoQ (open circles). Data are presented as mean ± SEM and were analyzed with two-way ANOVA with Sidak's multiple comparisons post-hoc test. N = 4–5 offspring/ n = 1 offspring per dam/group. *a.u.* arbitrary units, *MFI* mean fluorescence intensity

**Fig. 8:**
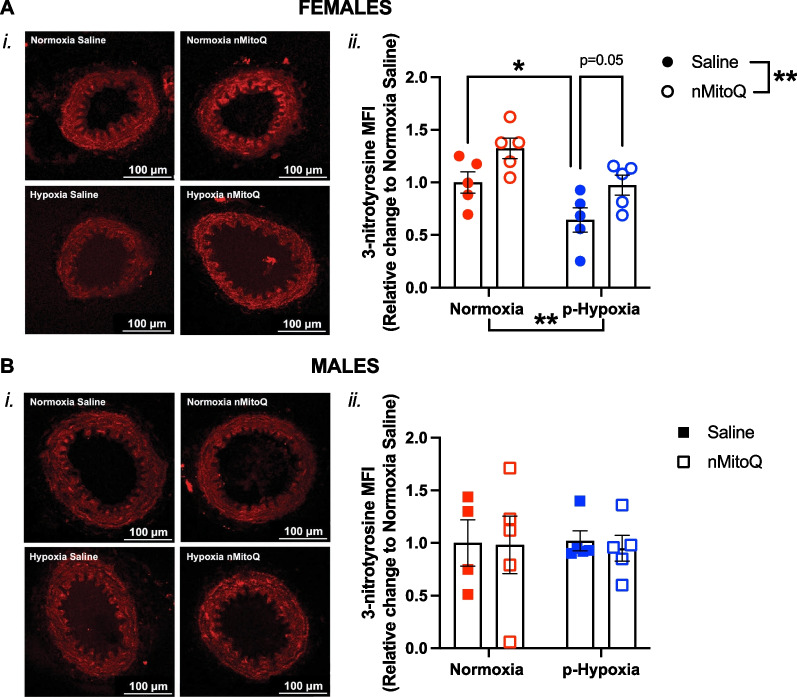
Detection of 3-nitrotyrosine in mesenteric arteries of adult offspring exposed to prenatal hypoxia. Representative confocal images (*i*) and quantitative analysis (*ii*) of 3-nitrotyrosine, an indirect measurement of peroxynitrite formation, in mesenteric arteries of female (**A**, circles) and male (**B**, squares) offspring exposed to normoxia (red) or hypoxia (blue; p-Hypoxia) during pregnancy and after receiving maternal treatment with saline (vehicle, closed circles) or nMitoQ (open circles). Data are presented as mean ± SEM and were analyzed with two-way ANOVA with Sidak's multiple comparisons post-hoc test (*p < 0.05; **p < 0.01). N = 4–5 offspring/ n = 1 offspring per dam/group. *a.u.* arbitrary units, *MFI* mean fluorescence intensity

**Fig. 9:**
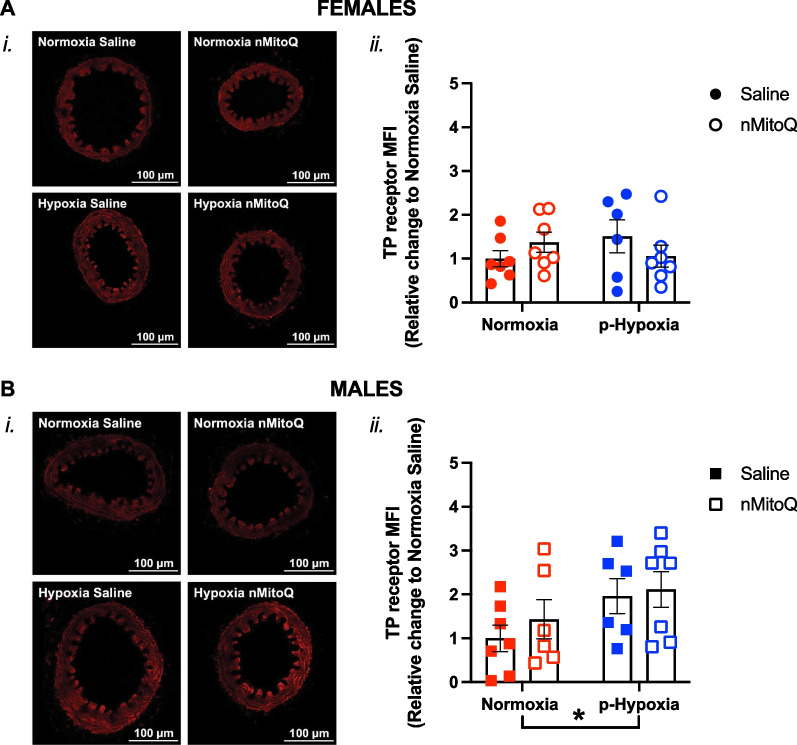
Expression of thromboxane prostanoid (TP) receptors in mesenteric arteries of adult offspring exposed to prenatal hypoxia. Representative confocal images (*i*) and quantitative analysis (*ii*) of TP receptor expression in mesenteric arteries of female (**A**, circles) and male (**B**, squares) offspring exposed to normoxia (red) or hypoxia (blue; p-Hypoxia) during pregnancy and after receiving maternal treatment with saline (vehicle, closed circles) or nMitoQ (open circles). Data are presented as mean ± SEM and were analyzed with two-way ANOVA with Sidak's multiple comparisons post-hoc test (*p < 0.05). N = 6–7 offspring/ n = 1 offspring per dam/group. *a.u.* arbitrary units, *MFI* mean fluorescence intensity

A two-way ANOVA with Sidak’s post-hoc analysis was used for assessment of the effect of prenatal hypoxia and sex (Fig. 1) or maternal nMitoQ treatment (Figs. 2, 3, 4, 5, 6, 7, 8, 9). Data are expressed as mean ± standard error of the mean (SEM). Outliers were tested using Grubb's test, and were removed from the final analysis if significant. For the wire myography data, the CCRCs were summarized using the negative logarithm of the half-maximal effective concentration (pEC_50_) and/or the area under curve (AUC). Data were analyzed using the GraphPad Prism 9.4.0 software (GraphPad, San Diego, USA), and a p ≤ 0.05 was considered statistically significant.

## Results

### Prenatal hypoxia increased vasoconstriction to U46619 in coronary and mesenteric arteries of the adult female offspring

Exposure to prenatal hypoxia increased vasoconstriction responses to TxA_2_ in both coronary (p = 0.0019; Fig. 1A) and mesenteric (p = 0.0132; Fig. 1B) arteries from the female offspring compared to female normoxia controls. In contrast, there was no significant impact of prenatal hypoxia on overall coronary and mesenteric artery vasoconstriction to U46619 in the male offspring, compared to male normoxia controls (Fig. 1A and B).

### Prenatal hypoxia increased vasoconstriction to U46619 in coronary arteries of the adult offspring, which was prevented by nMitoQ treatment in the females

In both male and female offspring, exposure to prenatal hypoxia shifted the U46619 curve to the left (Fig. 2A*i* and B*i*). In coronary arteries of the adult female offspring, exposure to prenatal hypoxia increased the sensitivity (pEC_50_; p = 0.0002) and overall vasoconstriction responses (AUC; p = 0.0007) to U46619 compared to female normoxia control offspring (Fig. 2A). Moreover, this increased vasoconstriction and sensitivity to U46619 in coronary arteries of the prenatal hypoxia exposed females was significantly reduced by maternal treatment with nMitoQ (p = 0.0267; Fig. 2A*ii* and *iii*). In coronary arteries of the male offspring, prenatal hypoxia exposure only increased the sensitivity to U46619, compared to male normoxia control offspring (p = 0.0112; Fig. 2B*ii*). However, this was not prevented by maternal nMitoQ treatment in coronary arteries of the male prenatal hypoxia offspring (Fig. 2B*ii* and iii).

### Prenatal hypoxia increased vasoconstriction to U46619 in mesenteric arteries of the female offspring via reduced NO modulation, which was prevented by maternal nMitoQ treatment

Similar to the coronary arteries, in mesenteric arteries from the adult female offspring, U46619-mediated vasoconstriction responses were also increased by exposure to prenatal hypoxia (p = 0.0072), which was prevented by maternal treatment with nMitoQ in the prenatal hypoxia females (p = 0.0470; Fig. 3A*iii*). However, there were no significant changes in sensitivity to U46619 by prenatal hypoxia in the females (Fig. 3A*ii*). In contrast, in mesenteric arteries of the male adult offspring, although there were no significant differences in overall U46619-mediated vasoconstriction responses (Fig. 3B*iii*), exposure to prenatal hypoxia increased the sensitivity to U46619 compared to normoxia control offspring (p = 0.0001), and this was prevented by maternal nMitoQ treatment (p = 0.0019; Fig. 3B*ii*). nMitoQ treatment also increased the sensitivity to U46619 in the normoxia male offspring compared to normoxia male offspring of saline treated dams (p < 0.0001; Fig. 3B*ii*).

Next, we assessed whether the increased vascular U46619 responses in the prenatal hypoxia offspring could be due to the reduced NO modulation in the mesenteric arteries. We observed that in mesenteric arteries of the female normoxia offspring, the pan-NOS inhibitor L-NAME increased U46619 responsiveness in both saline (p = 0.0030) and nMitoQ-treated (p = 0.0003) normoxia offspring (Fig. 4Aii), as well as prenatal hypoxia offspring that received maternal nMitoQ treatment (Fig. 4Bii), compared to control vessels without L-NAME. However, in mesenteric arteries from female offspring exposed to prenatal hypoxia and saline treatment, there was no significant effect of pre-incubation with L-NAME on vasoconstriction responses to U46619 (Fig. 4B). In contrast, in the male offspring, L-NAME increased U46619 vasoconstrictor responses in mesenteric arteries irrespective of prenatal exposure (normoxia saline: p = 0.0390; normoxia nMitoQ: p = 0.0167; p-hypoxia saline: p = 0.0002; p-hypoxia nMitoQ: p = 0.0028) compared to the control vessels without L-NAME (Fig. 5A and B).

### Prenatal hypoxia decreased mesenteric artery 3-nitrotyrosine levels in the females while increasing TP receptor expression in the male offspring

There was no significant impact of exposure to prenatal hypoxia or maternal nMitoQ treatment on mesenteric artery eNOS expression (Fig. 6A and B) or ROS levels (Fig. 7A and B) in the female or male offspring. However, in mesenteric arteries of the female offspring, 3-nitrotyrosine levels were lower after prenatal hypoxia exposure (p = 0.0499), which was prevented by nMitoQ treatment (Fig. 8A). No effects of prenatal hypoxia or maternal nMitoQ treatment on 3-nitrotyrosine levels were observed in mesenteric arteries of the male adult offspring (Fig. 8B). Neither prenatal hypoxia nor maternal nMitoQ treatment altered the TP receptor expression in mesenteric arteries from the female offspring (Fig. 9A). However, prenatal hypoxia exposure increased the TP receptor expression in mesenteric arteries from the male offspring (p = 0.0470, overall effect; Fig. 9B), without effects of maternal nMitoQ treatment.

## Discussion

In this study, we aimed to assess the impact and potential underlying mechanisms of prenatal hypoxia and maternal nMitoQ treatment on vascular TxA_2_-mediated constriction in male and female adult offspring. Interestingly, we observed sex-specific differences in the effect of prenatal hypoxia on offspring TxA_2_-mediated vasoconstriction; where adult female rats exposed to prenatal hypoxia showed a more severe phenotype in both coronary and mesenteric arteries, with higher responsiveness and sensitivity to TxA_2_, while prenatal hypoxia also impacted TxA_2_ responses (higher sensitivity only) in the male offspring, but this impact was less severe. Maternal treatment with nMitoQ prevented the prenatal hypoxia-induced effects on the increased vascular responses in coronary and mesenteric arteries from the female adult offspring, while in the male offspring, nMitoQ prevented the impact of prenatal hypoxia exposure in mesenteric, but not coronary, arteries. It is noteworthy that this phenotype induced by prenatal hypoxia was observed in two inherently different vascular beds, the coronary and mesenteric arteries.

The coronary arteries are essential for the maintenance of proper cardiac performance [42]. We recently demonstrated that exposure to prenatal hypoxia was associated with impaired endothelium-dependent vasodilation in the coronary arteries of both male and female adult offspring [17]. Our current data of increased TxA_2_ responses in the prenatal hypoxia offspring suggests that the coronary vasculature is highly susceptible to the impact of prenatal hypoxia. Intriguingly, we have previously shown that the cardiac recovery to I/R insult is reduced by prenatal hypoxia in the adult offspring, which was prevented by nMitoQ treatment [34, 43]. The coronary circulation is essential in reperfusion of the ischemic cardiac tissue and for cardiac recovery [44, 45]. Moreover, ischemia has been reported to stimulate TxA_2_ synthesis [46] and TxA_2_ may be involved in reperfusion injury, as TxA_2_ receptor blockade has been suggested as a mode of therapy for I/R damage [47]. Thus, it may be speculated that the increased TxA_2_ responses in the coronary arteries contribute to the impaired recovery to I/R insult due to prenatal hypoxia, and that nMitoQ treatment prevented this by improving coronary artery function; however, this remains to be addressed in future studies.

We observed that in adult females, exposure to prenatal hypoxia enhanced the sensitivity and the overall responsiveness to TxA_2_ in coronary arteries, as well as the overall TxA_2_ responses in mesenteric arteries, which was prevented by maternal nMitoQ treatment. In contrast, in the male offspring, exposure to prenatal hypoxia increased the TxA_2_-mediated sensitivity in both coronary and mesenteric arteries, however, maternal nMitoQ treatment only prevented this increased sensitivity in the mesenteric arteries. Similar to our current findings, a previous study (assessing only male offspring) reported an increased sensitivity to TxA_2_ in mesenteric arteries of adult male prenatal hypoxia exposed offspring [27]. However, to the best of our knowledge, this is the first study showing increased TxA_2_-mediated vasoconstriction as an effect of a complicated pregnancy in adult female offspring. TxA_2_ is important for platelet activation, but is also involved in vascular wall pathology, including impaired endothelium-dependent vasodilation, increased oxidant generation, and increased adhesion molecule expression, thus playing an important role in the local regulation of vascular tone in both physiological and pathological states [48, 49]. Notably, cardiovascular conditions such as hypertension are associated with increased TxA_2_ responsiveness [50]. Moreover, prenatal hypoxia has been shown to increase responsiveness to other vasoconstrictors. For example, adult male offspring exposed to prenatal hypoxia was shown to have increased responsiveness to phenylephrine in mesenteric [51] and renal interlobar arteries [52], to endothelin-1 in pulmonary arteries [53] and big endothelin-1 in mesenteric arteries [19], and to angiotensin II in middle cerebral arteries [54]. Taken together, this could imply that prenatal hypoxia induces a general increase in systemic vasoconstriction in the offspring, which could contribute to the development of hypertension and/or other cardiovascular diseases later in life. Of note, in humans, prenatal hypoxia exposure is associated with the development of hypertension in the adult offspring [14, 55]. In our rat model of prenatal hypoxia, we previously did not observe changes in blood pressure in the offspring at 7 months of age [31], but did see an increased blood pressure in the male offspring at 14 months of age [19]. Thus, the increased systemic vasoconstriction at 4 months of age could be a sign of a pre-hypertensive state, that is exacerbated by a second hit such as aging.

We used the mesenteric arteries to address the potential mechanistic pathways by which exposure to prenatal hypoxia increased TxA_2_–mediated vasoconstriction in the adult offspring. In the female prenatal hypoxia offspring, the increased TxA_2_ responsiveness appeared to be due to a reduction in NO modulation of vasoconstriction. Moreover, this reduction in NO modulation was prevented by maternal nMitoQ treatment. NO modulates vascular tone by inducing smooth muscle relaxation thereby antagonizing the smooth muscle constriction induced by TxA_2_. NO also mediates flow-mediated vasodilation, counteracts vascular stiffness and lowers blood pressure, thus, NO is an important modulator of blood flow, blood pressure, and vascular tone [56]. In the vascular system, NO is mainly produced in by the predominant NOS isoform, eNOS [57]. Thus, changes in NO modulation of vasoconstriction could be due to oxidative stress (i.e. scavenging of NO by superoxide) or changes in NOS expression/activity. However, no changes in eNOS expression were observed between our groups. Oxidative stress, i.e. the overproduction of reactive oxygen species (ROS), such as superoxide, is involved in many pathological processes leading to impaired vascular function [58]. The balance between superoxide and NO is important for vascular homeostasis as superoxide reacts rapidly with NO, thereby reducing the bioactivity of NO and producing peroxynitrite, a strong and short-lived oxidant that can nitrosylate cellular proteins and lipoproteins [23]. The stable product of nitration of tyrosine residues by peroxynitrite, 3-nitrotyrosine, has been used as a marker of peroxynitrite production [59, 60]. In contrast to our expectations, we did not observed differences in ROS levels in mesenteric arteries between our groups, suggesting the reduced NO modulation was not due to oxidative stress. However, we did observe a reduction in peroxynitrite production in mesenteric arteries of the female prenatal hypoxia offspring. While these data may seem conflicting, they could reflect a general reduction in endogenous NO bioavailability (leading to less peroxynitrite production) due to other mechanisms, such as reduced NOS activity. NOS activity can be affected by various mechanisms not associated with oxidative stress, such as reduced intracellular levels of tetrahydrobiopterin (BH_4_), an important cofactor [61], reduction on the substrate L-arginine levels by arginase [62], phosphorylation of the threonine 495 (Thr^495^) residue [20], reduced affinity to calmodulin [63], and interaction with caveolin [64]. Thus, our data suggest that in the females, the reduced NO modulation of vasoconstriction is mediated by reduced NO bioavailability, is not associated with oxidative stress, and may be mediated via other pathways impacting NOS activity.

In contrast to the females, mesenteric and coronary arteries of the male offspring, while also showing an increased sensitivity to TxA_2_ compared to normoxia controls, were significantly less impacted by prenatal hypoxia. Intriguingly, there were no differences in NO modulation to vasoconstriction, as had been observed in the females. However, the increased sensitivity to TxA_2_ vasoconstriction in the male hypoxia offspring compared to normoxia control offspring could be explained by the increased TP receptor expression promoted by prenatal hypoxia exposure in males. The TP receptor is a G-protein coupled receptor, and its activation requires phospholipase C coupling, induces inositol trisphosphate biosynthesis and Rho kinase activation [65], leading to vasoconstriction. Moreover, increased activation of the TP receptor plays an important role in the pathophysiology of cardiovascular diseases [66]. These findings reinforce the sex-specific impact that prenatal hypoxia has on vascular function in the offspring.

Taken together, the increased TxA_2_ responses were more pronounced in females and there were sex-specific differences in the molecular mechanisms for these changes. In the female offspring, the increased responsiveness to TxA_2_ appeared to be due to a reduced NO modulation to constriction. While in males the increased response to TxA_2_ appeared due to an increased expression on TP receptors. Interestingly, differences in mechanisms leading to similar overall impacts between males and female offspring exposed to prenatal hypoxia is something we have previously reported [17, 19, 31–34]. These findings together with our previous work emphasize the need for precision (sex-specific) medicine approaches.

As placental oxidative stress is thought to be one of the key consequences of prenatal hypoxia, we have been studying the effects of a maternal treatment in pregnancy that targets placental oxidative stress. We have used a nanoparticle-encapsulated mitochondrial antioxidant (nMitoQ), which, due to its size and charge, is primarily taken up by the placenta and thus avoids off-target effects on the fetus [36, 37]. Our current data show that treating the placenta during pregnancy with nMitoQ could be protective against the impact of prenatal hypoxia on vascular TxA_2_ responses in both the male and female adult offspring. In line with these findings, it was previously shown that maternal nMitoQ treatment reduced the sensitivity to phenylephrine in mesenteric arteries exposed to prenatal hypoxia [31]. We also demonstrated that maternal nMitoQ treatment prevented the impaired cardiac recovery to I/R insult in both male and female offspring [34]. Noteworthy, maternal nMitoQ treatment increased the sensitivity to TxA_2_ in mesenteric arteries of males born from normoxic control pregnancies, supporting what has been observed before [32–34], that maternal treatments and interventions should be restricted to those presenting with pregnancy complications. Thus, our study emphasises the impact that the placenta has on prenatal programming of offspring (cardio)vascular disease, and the beneficial effects of placenta-targeted interventions in the improvement of cardiovascular function in offspring exposed to unfavorable in utero environment.

### Perspectives and significance

Prenatal hypoxia is a common consequence of pregnancy complications and could thus impact up to 30% of all pregnancies [67]. CVDs are a major cause of mortality and an increasing health problem worldwide. Pregnancy complications such as prenatal hypoxia increase the risk of cardiovascular disease in the offspring later in life, but also offer a window of opportunity for early intervention. Our current data adds to the literature linking prenatal hypoxia to impaired cardiovascular outcomes in the offspring. Importantly, our findings show that this can be prevented by an early (prenatal) intervention that targets placental oxidative stress, supporting the concept that the placenta is at the source of this problem and indicating the placenta may be a successful target for future therapeutic developments. In addition, females are still severely underrepresented in basic and clinical research studies, resulting in a skewed view on the impact and mechanisms of many health conditions. Here, we show that females were more impacted, and that the mechanisms were sex-specific, highlighting the need to include both males and females in research studies to address these potential sex differences, as well as the need for sex-specific therapies later in life.

## Conclusion

In summary, in the current study, we showed that prenatal hypoxia, a common pregnancy complication, increases the vasoconstrictor responses to TxA_2_ in two different arterial beds, and primarily in the female adult offspring. This appeared to be due to a reduction in NO modulation of constriction in the females, while in the males this appeared due to increased TP receptors. Moreover, by increasing NO modulation to suppress vasoconstriction, maternal treatment with the placental antioxidant nMitoQ prevented the enhanced vasoconstriction in female offspring exposed to prenatal hypoxia. Our data suggest that prenatal hypoxia impairs systemic vascular TxA_2_ vasoconstrictor capacity in the offspring in a sex-specific manner, and that the mechanisms differ between sexes, thus sex differences need to be considered when developing precision therapeutic strategies for those at risk due to their prenatal environment. In addition, our data provides evidence that the environment experienced during critical, sensitive periods of fetal development can directly influence long-term cardiovascular health.

## Data Availability

The datasets used and/or analyzed during the current study are available from the corresponding author on reasonable request.
